# A Case of Chronic Pneumonia Following Inflammatory Rheumatism

**Published:** 1854-05

**Authors:** O. C. Gibbs

**Affiliations:** Perry, Lake Co. Ohio


*A case of Chronic Pneumonia following Inflammatory Rheumatism.* By O. C. Gibbs, M. D., Perry, Lake Co. Ohio.

Chronic pneumonia is doubtless a rare disease, whether considered as occurring idiopathically, or as a sequel of the acute form of pneumonic inflammation. That it may occur as a sequel of rheumatic inflammation I was not aware, until the following case came under my observation.

January 8th, 1850, I was consulted by E. Rawdon, aged 25 years, a muscular farm laborer, for a gradually increasing ailment of several weeks' duration. His symptoms were a slight cough, without pain in the chest sufficient to occasion complaint, considerable dyspnœa, which was much increased by exertion, a bluish paleness of the countenance, the leaden appearance of which was increased by exertion and exposure to cold, and an increased frequency of pulse, with more decided febrile symptoms towards evening. The appetite was impaired, though by no means lost, and he had strength only to walk about his apartment or ride in an easy carriage.

Auscultation revealed an entire absence of the respiratory murmur, and percussion gave entire dulness throughout the whole of the right lung, excepting the upper third.

Several weeks (the exact length of time is not remembered) before consulting me, he had been attacked with and treated for what his physician called a rheumatic inflammation of the right knee joint. This difficulty very soon disappeared, simultaneous with which the dyspnœa and slight cough manifested themselves, with slight pain at first, and the other symptoms above enumerated were gradually superadded.

His medical adviser, though a very good physician, being unacquainted with the art of physical diagnosis, was evidently at a loss to know how to account for the symptoms, and this diagnostic indecision found a counterpart in the lack of therapeutic efficiency.

The following treatment was ordered:

℞. Tinct. Cimicifugæ, f.℥vj.

Potassii Iodid., ʒj.

M. Dose, a teaspoonful twice a day, morning and noon, and one of the following pills at bedtime:

℞. Hydrarg. Iodid., gr. xv.

Ant. et pot. Tart., gr. v.

Ext. Hyoscyam., gr. l.

Mix ; and make into thirty pills.

In addition to the above treatment, a large blister was applied to the affected side, and the body ordered to be washed every night in whiskey and water.

At the end of a week the gums became tender, after which the iodide of mercury was given only as was necessary to maintain undiminished the slight mercurial impression. The antimony and hyoscyamus were, however, continued at bedtime, and the iodide of potassium as directed in the formula given above. Blisters were continued at weekly intervals to the affected side of the chest. Coincident with the manifestation of the mercurial impression, the expectoration, from being very slight, became quite profuse, muco-purulent in character, and more or less streaked with blood. This change in the character and quantity of the expectoration very much alarmed the patient and his friends, though accompanied with evidences of improvement.

The mercurial impression was kept up for ten weeks, by an occasional pill of iodide of mercury, aided by mercurial inunction to the chest. During the same time, the iodide of potassium was given, and blisters repeated at weekly intervals, when the patient was discharged; but in consequence of some remaining dulness upon percussion, the mixture as given above, was advised to be continued for a few weeks longer, with antimonial counter irritation to the chest.

The patient living nearly twenty miles from my residence, was not seen again for more than a year, at which time he seemed as healthy as ever, and the chest, on examination, gave no evidences of previous disease.

In pneumonic hepatization, whether resulting from acute or chronic inflammation, so far as my experience goes, no internal medicines equal in efficacy the combined action of mercury and iodine ; aided, as all medicines should be in such cases, by repeated blisters.

